# Confounding by Repetitive Elements and CpG Islands Does Not Explain the Association between Hypomethylation and Genomic Instability

**DOI:** 10.1371/journal.pgen.1003333

**Published:** 2013-02-28

**Authors:** R. Alan Harris, Chad Shaw, Jian Li, Sau Wai Cheung, Cristian Coarfa, Mira Jeong, Margaret A. Goodell, Lisa D. White, Ankita Patel, Sung-Hae Kang, A. Craig Chinault, Tomasz Gambin, Anna Gambin, James R. Lupski, Aleksandar Milosavljevic

**Affiliations:** 1 Bioinformatics Research Laboratory, Epigenome Center, Baylor College of Medicine, Houston, Texas, United States of America; 2 Department of Molecular and Human Genetics, Baylor College of Medicine, Houston, Texas, United States of America; 3 Program in Structural and Computational Biology and Molecular Biophysics, Baylor College of Medicine, Houston, Texas, United States of America; 4 Institute of Computer Science, Warsaw University of Technology, Warsaw, Poland; 5 Institute of Informatics, Warsaw University, Warsaw, Poland; 6 Department of Pediatrics, Baylor College of Medicine, Houston, Texas, United States of America; 7 Texas Children's Hospital, Houston, Texas, United States of America; HudsonAlpha Institute for Biotechnology, United States of America

In our recent article [1], we reported an association between hypomethylation and genomic instability. A comment by Watson et al. [2] re-analyzes the data and claims that our findings may represent an artifact. We extend the methodological framework for analyzing copy number variants (CNVs) in the context of potential confounding factors to address the issues raised in the comment and to further research in this growing area of genomic science.

Watson et al. argue that the association we reported between hypomethylation of genomic DNA—determined from sperm methylomes [3]—and the density of CNVs can be explained by a combined confounding effect of known correlates of CNVs, namely repetitive elements and CpG islands. To support their argument, the authors eliminate many genomic regions providing a variety of justifications for why these regions create “spurious association”. Once the regions have been removed from the genome, Watson et al. claim the association between hypomethylation and genomic instability disappears.

We would first like to point out that the goal of our study was not to ignore potential relevance of these other factors—such as repetitive elements and CpG islands—but instead to broaden the scope of inquiry to examine possible additional explanatory power of hypomethylation. In deference to Watson et al., we initiated a re-analysis that systematically examined the purported confounding factors. Rather than pursuing the data exclusion approach used by Watson et al., we applied the standard multiple regression approach. The regression methods control for confounding without discarding data or otherwise biasing the inquiry to particular genomic sub-regions. Specifically, we first asked if the variables brought up by Watson et al. (LINE, SINE, LTR, Satellite, and CpG island content) individually or in combination explain the association between hypomethylation and CNV counts within 100-Kbp windows tiling the genome. In addition, using the Akaike information criterion (AIC) we measured the explanatory power of each of the six variables beyond the explanatory power of the other five.

We first applied the Negative Binomial regression model [4], because it is commonly used for overdispersed variables and is a well-accepted and robust method for count data such as CNV counts. We applied the same method to all five sample sets brought up by Watson et al.—HapMap270 [5], HapMap450 [6], WTCCC [7], Schizophrenia Cases, and Schizophrenia Controls [8]. As documented in Tables S1 and S2, in all five sample sets hypomethylation remained highly significantly associated with CNV density after correction for all of the “confounders” individually and in combination. As illustrated in Figure 1, as measured by AIC, methylation was more predictive of CNV counts per 100-Kbp window by an order of magnitude than any other factor.

**Figure 1:**
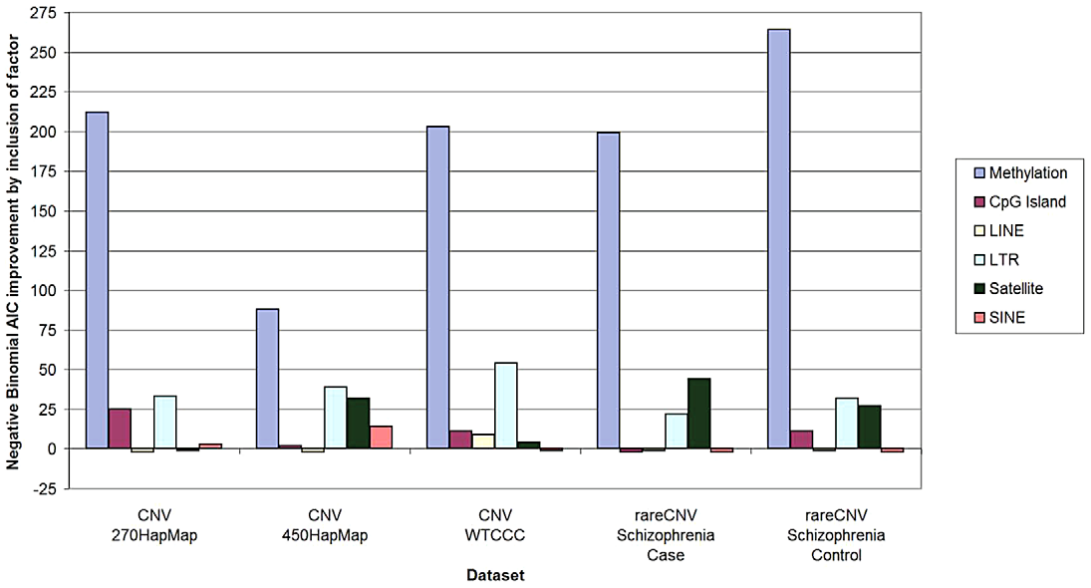
Predictive power of methylation and other genomic factors for CNV counts. Predictive power of methylation, CpG island content, and repetitive element content (LINE, SINE, LTR, and Satellites) was measured using Akaike information criterion (AIC). For all five datasets, negative binomial regression was performed using all six factors and all six combinations of five factors (one factor being removed at a time). The *y*-axis represents the predictive power of a factor, as measured by the improvement of the AIC score based on all six factors relative to the AIC score without the factor. Note that this method measures predictive power of a factor after correction for any potential confounding due to other factors. (The detailed calculations and input data are in Supporting Information.)

To examine robustness of our analyses with respect to modeling assumptions, we repeated the same analysis by applying the more widely used Poisson and linear regression models. All three models gave consistent results for all confounders in all five sample sets. (Table S2 describes regression models, output, and the input data extracted from our original paper sufficient to run a statistical program such as R to obtain the output.) We also employed zero-inflated negative binomial and Poisson regression models, and found completely concordant results. We therefore conclude that the assertions regarding “confounding” are not consistent with the data available. This is likely because Watson et al. resorted to an idiosyncratic selective data elimination procedure rather than pursuing a more standard statistical approach.

Second, Watson et al. bring up mappability of reads as a confounding factor while failing to mention that the original paper [1] considered and—using bisulfite sequencing data from embryonic stem cell H1 as a control—ruled out ascertainment biases due to read mappability: “We next examined the difference in methylation levels between sperm and H1. As illustrated in Figure S16, the difference shows even stronger association with structural mutability than the absolute methylation levels in sperm. This result rules out possible ascertainment biases due to low mappability of sequencing reads in potentially unstable and repetitive hypomethylated regions. It also suggests that structural mutability is associated with germline-specific hypomethylation.” Also quoting from our article: “We found significant negative correlation between the methylation scores in sperm and the heterozygosity rates (CNVs from 400 MGL samples: r≈−0.15, p≈10^−9^; CNVs from 270 HapMap samples: r≈−0.20, p≈10^−10^). In contrast, no significant correlation between the H1 methylation scores and the CNV heterozygosity rates was detected” [1].

Third, Watson et al. argue that the higher mode with zero scores for the Methylation Index (MI = 0) is likely an artifact due to small SNP and CpG counts. In this context it is surprising that Watson et al. fail to mention that our article considered, examined, and ruled out this possibility: “One could expect that if the windows with MI = 0 were due to low probing density, the windows within the higher mode would have fewer SNPs or CpGs. However, we examined potential biases in MI estimation due to variations in the number of SNPs, CpGs, read coverage (Figure S6CD), or sampling events (Figure S7BD) and found no significant difference between the two modes, ruling out the possibility that the two modes may be explained by variation in mappability or shallow sampling. In addition, a simulation experiment showed that the statistical variance of methylation estimates due to CpG sampling of windows with MI = 0 was a relatively small fraction of biological variance in methylation observed between the two sperm methylomes (Figure S8). We therefore hypothesize that the higher mode may either indicate hypomethylation specific to the female germline, given that male and female germline methylation patterns are highly dimorphic [47], or may be due to other germline hypomethylation detected by MI that is absent from sperm.”

Fourth, the “confounders” brought up by Watson et al. do not influence genomic instability independently of the methylation state and therefore do not meet the common definition of confounding [9]. In the specific case of CpG islands, the striking pattern where hypomethylated CpG islands are enriched in unstable regions (Figure 2A and 2B in Watson et al. [2]) would in fact be expected to occur if hypomethylation were mechanistically linked to genomic instability. Watson et al. ignore this possibility without sound justification while claiming that this pattern somehow provides evidence *against* any connection of hypomethylation and genomic instability.

Fifth, contrary to what Watson et al. claim, our article does not state that hypomethylation plays a causative role in genomic instability. Specifically, in the discussion section of our article we state three possible mechanistic explanations for the observed association: DNA break–inducing germline-specific demethylation during embryogenesis; mutagenic effects of germline-specific gene expression in hypomethylated loci; and mutagenic effects of transcription factor binding to hypomethylated loci.

In summary, we thank Watson et al. for their efforts and further examination of our reported observations. Nevertheless, we find that the arguments put forward in the comment do not diminish the strength of our reported findings. Specifically, our analyses of the confounding factors suggested by Watson et al. do not diminish the contention that genomic correlates may provide only a partial explanation for the hotspots of genomic instability. Thus, broadening inquiry to also include the epigenome may be warranted.

## Supporting Information

Table S1 CNV counts and values for methylation and other genomic factors for 100-Kbp windows tiling the hg18 assembly of the human genome.
(ZIP)

Table S2 Regression analysis of the predictive power of methylation and other genomic factors for CNV counts.
(XLS)

## References

[1] Li J, Harris RA, Cheung SW, Coarfa C, Jeong M, et al (2012) Genomic hypomethylation in the human germline associates with selective structural mutability in the human genome. PLoS Genet 8 (5) e1002692 doi:10.1371/journal.pgen.1002692 22615578 10.1371/journal.pgen.1002692 PMC3355074

[2] Watson CT, Garg P, Sharp AJ (2013) Comment on “Genomic hypomethylation in the human germline associates with selective structural mutability in the human genome”. PLoS Genet 9: e1003332 doi:10.1371/journal.pgen.1003332 23468658 10.1371/journal.pgen.1003332 PMC3585013

[3] Molaro A, Hodges E, Fang F, Song Q, McCombie WR, et al (2011) Sperm methylation profiles reveal features of epigenetic inheritance and evolution in primates. Cell 146 (6) 1029–1041. 21925323 10.1016/j.cell.2011.08.016 PMC3205962

[4] Hilbe J. (2011) Negative binomial regression. Cambridge, UK: Cambridge University Press. p. 553.

[5] McCarroll SA, Kuruvilla FG, Korn JM, Cawley S, Nemesh J, et al (2008) Integrated detection and population-genetic analysis of SNPs and copy number variation. Nat Genet 40 (10) 1166–1174. 18776908 10.1038/ng.238

[6] Conrad DF, Pinto D, Redon R, Feuk L, Gokcumen O, et al (2010) Origins and functional impact of copy number variation in the human genome. Nature 464 (7289) 704–712. 19812545 10.1038/nature08516 PMC3330748

[7] The Wellcome Trust Case Control Consortium (2010) Genome-wide association study of CNVs in 16,000 cases of eight common diseases and 3,000 shared controls. Nature 464 (7289) 713–720. 20360734 10.1038/nature08979 PMC2892339

[8] The International Schizophrenia Consortium (2008) Rare chromosomal deletions and duplications increase risk of schizophrenia. Nature 455 (7210) 237–241. 18668038 10.1038/nature07239 PMC3912847

[9] Pearl J (2000) Causality: models, reasoning, and inference. Cambridge, UK: Cambridge University Press. 384 p.

